# Determinants of primary healthcare physicians’ knowledge of diabetic kidney disease

**DOI:** 10.4102/phcfm.v18i1.5322

**Published:** 2026-04-29

**Authors:** Wafaa Fadili

**Affiliations:** 1 Department of Nephrology, UHC Mohamed VI, Faculty of Medicine and Pharmacy, Cadi Ayyad University, Marrakesh, Morocco

**Keywords:** Diabetic kidney disease, primary practitioners, knowledge evaluation, screening, management, referral

## Abstract

**Background:**

Early identification of diabetic kidney disease (DKD) is a real means to prevent the progression of kidney disease. The involvement of primary practitioners in screening programmes is particularly needed as most patients with diabetes are managed in primary care.

**Aim:**

This study aims to assess primary practitioners’ knowledge regarding screening, referral and management guidelines of patients with diabetes.

**Setting:**

Routine clinical setting in the region of Marrakesh.

**Methods:**

This was a cross-sectional study including primary practitioners in the region of Marrakesh between May and June 2024. A 16-item questionnaire was developed to evaluate their knowledge about screening, referral and management of DKD. Multivariate analysis was performed to identify factors associated with knowledge levels.

**Results:**

Of the 295 general practitioners solicited for the study, 100 physicians agreed to participate, thus translating to a response rate of 33.9%. Most participants (61%) showed high levels of adherence to DKD guidelines, but they presented a substantial gap in knowledge regarding the optimum screening time of DKD in patients with type 1 diabetes, the ideal screening test for albuminuria, the recommended interval for the routine surveillance, the annual screening tests, the therapeutic target of hemoglobin A1c (HbA1c), the indications of Sodium-Glucose Cotransporter-2 inhibitors (SGLT2i) and the timing of referral to a nephrologist. Higher knowledge scores were observed among physicians who received training in diabetes management.

**Conclusion:**

This study showed varying degrees of knowledge across different aspects of DKD management in primary care. Therefore, renal health programmes should prioritise enhancing the involvement and the regular training of primary practitioners.

**Contribution:**

This study emphasises the need for ongoing DKD training among primary healthcare practitioners to enhance their knowledge and improve the early management of patients with DKD.

## Introduction

Diabetes mellitus is a real global health problem. The estimated global population of adults with diabetes was 588.7 million in 2024, and the International Diabetes Federation (IDF) in the 11th edition of its Diabetes Atlas has projected that by the year 2050, about 852.5 million adults aged 20–79 years around the world will be living with diabetes mellitus.^1^ Among IDF regions, the African (AFR) and Middle East and North Africa (MENA) regions are projected to experience the largest relative increases in the number of people with diabetes. Morocco is one of the countries of the IDF MENA region and accounted for 9.7% of diabetes in adults in 2021.^2^ The increase in the prevalence of diabetes has been paralleled by an increase in the occurrence of its chronic complications, such as diabetic kidney disease (DKD). In 2011, it was estimated that diabetes was one of the main causes of chronic kidney disease (CKD) in Morocco.^3^

Diabetic kidney disease is the leading cause of CKD as well as end-stage renal disease (ESRD)^4^ and contributes considerably to the additional healthcare costs of the management of people with diabetes.^5^ Therefore, early diagnosis and treatment of DKD are critical to prevent kidney disease progression and other complications of diabetes.^6^ The detection of a positive albuminuria identifies the early stages of DKD,^6^ but screening relies on a dual assessment of urinary albumin (using a spot urinary albumin-to-creatinine ratio [UACR]) and estimated glomerular filtration rate (eGFR).^7^

For most people, DKD is asymptomatic and is often diagnosed through routine screening.^8^ Annual DKD screening is recommended by both the American Diabetes Association (ADA) and Kidney Disease Improving Global Outcomes (KDIGO) guidelines,^9,10^ and should start at diagnosis of type 2 diabetes and 5 years after diagnosis of type 1 diabetes.^9,10^ Based on these recommendations, DKD is diagnosed in the case of persistently positive albuminuria (≥ 30 mg/g) and/or persistently reduced eGFR (˂ 60 mL/min/1.73 m^2^) for more than 3 months.^9,10^

As most patients with diabetes are managed in primary care,^11^ primary care providers play an important role in the early detection of DKD.

The role of primary practitioners is firstly, to make a timely diagnosis of DKD through early screening of patients with diabetes and secondly, to delay the progression of DKD through efficacious glycaemic and blood pressure control, Renin-Angiotensin system (RAAS) blockade and SGLT2 inhibition in addition to health promotion and comorbidities management.^12^

However, despite international guidelines for screening and managing DKD, early detection and treatment of DKD in primary care settings is not performed optimally in routine clinical settings.^13,14^

A gap between ideal and actual care may be because of multiple factors, including a lack of knowledge about the disease itself, the fear of frightening patients with the announcement of CKD diagnosis, high workload or unfamiliarity with current guidelines.^6,7^

Therefore, it is important to enhance the effectiveness of the management of patients with diabetes and the prevention of the development of complications.^15^

As primary care is usually the first point of contact for most patients with early-stage CKD,^16^ assessing the degree of adherence by the primary healthcare physicians to current guidelines that promote early detection, prevention and treatment of DKD is recommended.

### Objective of the study

This study aimed to evaluate primary healthcare physicians’ knowledge regarding screening, referral and management guidelines of DKD in patients with diabetes.

## Research methodology and design

### Study design

A cross-sectional study was conducted between May 2024 and June 2024, and all general practitioners working at primary care centres of the region of Marrakesh were included.

### Population, sample and sampling

All primary healthcare practitioners working in the private and public sectors in the region of Marrakesh were contacted by phone and were informed about the study. Physicians who refused to participate in the survey were excluded.

### Data collection

Participants were asked to complete a self-administered questionnaire. The questionnaire contained 16 items and was designed to assess practitioners’ knowledge of screening, diagnosis and management of DKD. The questions were constructed by consulting relevant ADA and KDIGO guidelines on DKD.^8^ This test included single-choice questions and consisted of two sections: section 1 (demographics of the physicians, length of professional experience and training in diabetes management), section 2 (knowledge level of guidelines about DKD screening, referral and treatment) (Appendix 1). A panel of experts, including six consultants in nephrology, reviewed the questionnaire and completed an online content validation form.^17^ The questionnaire’s reliability was assessed using the computation of Cronbach’s alpha coefficient. A reliability index of 0.88 was obtained.

An online form of the questionnaire was created using Google Forms and was sent to the participants via email or work-related WhatsApp groups. The practitioners who agreed to participate and completed the questionnaire were recruited into the study.

Each item of the questionnaire was assigned a score of 1 if the response was correct, and a total score was provided by adding all responses. The median value of the total scores of all participants’ responses to the questionnaire was utilised as a cut-off point to classify each physician as having low or high knowledge concerning DKD management, screening and referral.^15^

### Statistical analysis

Data were analysed using Statistical Package for Social Sciences (SPSS Inc, Chicago, US) (26.0). Participants’ demographic and professional characteristics were summarised for categorical variables as frequency with percentage, and for continuous variables as means ± standard deviation (s.d.) or median (minimum [min] – maximum [max]). In bivariate analysis, qualitative variables were compared using the Chi-square test or Fisher’s exact test when the expected frequencies were less than 5.

In multivariate analysis, to identify factors associated with physicians’ knowledge of DKD guideline practice, a logistic regression model was used, and the results were expressed as odds ratios (OR). A *p*-value of ˂ 0.05 was statistically significant.

### Ethical considerations

Ethical clearance to conduct this study was obtained from the Ethics Review Committee of Faculty of Medicine and Pharmacy of Marrakesh (No. 5/2025). The objectives of the study were explained to the participants, and voluntary informed verbal consent was obtained from all participants. All information collected from the participants was kept confidential and was carried out in accordance with the Declaration of Helsinki.

## Results

### Response rate

Of the 295 primary healthcare providers practising in the region of Marrakesh, 100 physicians agreed to participate in the survey, yielding a response rate of 33.9%.

### Demographic findings

The demographic characteristics of the 100 primary physicians who agreed to participate are shown in Table 1.

**TABLE 1:** Demographic characteristics of participants.

Variables	Values (%)
**Gender**	
Female	60
Male	40
**Activity sector**	
Public	54
Private	46
**Experience length (years)**	
˂ 10	57
≥ 10	43
**Training in diabetes management**	
Yes	48
No	52

Notes: Age (years old, mean ± s.d.) = 47.57 ± 10.75. The total number of participants was 100. There was a female predominance and the majority had less than 10 years of experience and did not receive training in diabetes management.

The mean age of the study population was 45.57 (s.d. ± 10.75 years) with 60% being female (*n* = 60). The majority of the participants had less than 10 years of experience (57%), and 81% of them reported following more than 10 patients with diabetes per month. Forty-eight participants (48%) received training in diabetes management.

### Knowledge of screening, referral and management of diabetic kidney disease

The median score of correct answers was 8 (2–13), and 61% of participants had a score higher than 8.

The first part of the assessment was about diagnostic criteria of DKD, the timing of first screening and the frequency of screening. Within the project sample, 64% of the participants reported that the diagnosis of DKD was made in the case of a positive albuminuria with or without a reduced glomerular filtration rate (GFR), and that the usual start of screening of DKD should be performed at the time of diagnosis in the case of type 2 diabetes (77%) and 5 years after diagnosis in the case of type 1 diabetes (47%). Regarding the method used to screen for DKD, 34% of the participants chose the UACR, and 77% of them indicated that a positive UACR was defined by a result of 30 mg/g. Only 48% of the participants considered that a positive urine albuminuria screening should be repeated within 3–6 months of the first screen, and that the annual screening should include albuminuria (97% of the participants) and GFR (51% of the participants) (Table 2).

**TABLE 2:** Participants’ knowledge level regarding screening, management and referral of diabetic kidney disease.

Statements	Responses (*n*)	Responses (%)
**Diagnostic criteria of DKD:**		
Albuminuria ± reduced GFR	64	64
Oedematous state	22	22
Positive haematuria	13	13
No idea	1	1
**When should patients with type 2 diabetes first be screened for DKD:**		
At the time of diagnosis of diabetes	77	77
5 years after the onset of the disease	23	23
When the patient develops clinical signs of the disease	0	0
No idea	0	0
**When should patients with type 1 diabetes first be screened for DKD:**		
At the time of diagnosis of diabetes	53	53
5 years after the onset of the disease	47	47
When the patient develops clinical signs of the disease	0	0
No idea	0	0
**The diagnosis of DKD is made if a diabetic patient presents:**		
A single positive test of albuminuria	34	34
Two or three positive tests of albuminuria at an interval of 3–6 months	48	48
A haematuria	13	13
No idea	5	5
**The recommended way to screen for albuminuria:**		
24-h urine for albumin collection	63	63
Urine dipstick	3	3
Spot urinary albumin-to-creatinine ratio (UACR)	34	34
No idea	0	0
**The minimal threshold for a positive albuminuria corresponds to:**		
UACR ≥ 30 mg/g	77	77
UACR > 150 mg/g	12	12
UACR > 300 mg/g	11	11
No idea	0	0
**The recommended nephroprotective treatment of DKD:**		
Calcic inhibitors	10	10
ACE or ARB	70	70
Beta-blockers	5	5
No idea	15	15
**Time of referral to a nephrologist:**		
As soon as a positive albuminuria is diagnosed	54	54
When GFR is <30 mL/min on two consecutive visits or in case of a rapid decline of GFR	46	46
All above	0	0
No idea	0	0
**The recommended target of arterial pressure in the case of a positive albuminuria:**		
< 140/90 mmHg	38	38
< 130/80 mmHg	61	61
< 150/90 mmHg	0	0
No idea	1	1
**The recommended target of glycated haemoglobin in the case of CKD**		
≤ 6.5%	11	11
≤ 7%	48	48
≤ 8%	37	37
No idea	4	4
**Albuminuria should be screened annually:**		
True	97	97
False	3	3
**GFR should be screened annually:**		
True	51	51
False	49	49
**Metformin is contraindicated if GFR is:**		
< 45 mL/min per 1.73 m^2^	28	28
< 60 mL/min per 1.73 m^2^	10	10
< 30 mL/min per 1.73 m^2^	62	62
No idea	0	0
**SGLT2 inhibitors are recommended in:**		
Patients with type 2 diabetes, CKD and GFR ˂ 20 mL/min per 1.73 m^2^	31	31
Patients with type 1 diabetes	10	10
Patients with type 2 diabetes, CKD and GFR ≥ 20 mL/min per 1.73 m^2^	33	33
No idea	26	26

Note: Percentages are calculated from the total number of participants in this group (100 participants).

DKD, diabetic kidney disease; GFR, glomerular filtration rate, CKD, chronic kidney disease; SGLT2, Sodium-Glucose Cotransporter-2; ACE, angiotensin-converting enzyme; ARB, angiotensin receptor blocker.

The second part of the evaluation concerned the management of DKD. When the physicians were asked about the first-line anti-hypertensive drugs in the case of DKD, 70% of the participants reported that they prescribed angiotensin-converting enzyme (ACE) or angiotensin receptor blocker (ARB), and 61% indicated that the recommended target of arterial pressure was < 130/80 mmHg. Upon asking the physicians about HbA_1c_ recommended target, 48% reported that glycated haemoglobin should be < 7% (Table 2).

Considering the use of metformin, 62% of the participants knew that metformin was contraindicated in patients with CKD and GFR < 30 mL/min per 1.73 m^2^, and 33% of the participants reported that SGLT2 inhibitors were recommended in patients with type 2 diabetes, CKD and GFR ≥ 20 mL/min per 1.73 m^2^.

Regarding the timing of referral to a nephrologist, 46% of the participants answered that this referral should be at the time when GFR was < 30 mL/min per 1.73 m^2^ on two consecutive visits or if there was a rapid decline of GFR (Table 2).

In bivariate analysis, higher levels were found among practitioners over age 50 years and who reported attending training in diabetes management (*p* ˂ 0.001) (Table 3).

**TABLE 3:** Comparison of participants’ characteristics based on their level of knowledge in bivariate and multivariate analysis.

Characteristics	Bivariate analysis			Multivariate analysis		
	High knowledge level	Low knowledge level	*p*-value	Odds ratio	95%CI	*p*-value
**Age (years)**			0.034	2.427	0.894–6.589	0.082
≥ 50 years old (%) (ref)	25	8	-	-	-	-
˂ 50 years old (%)	36	31	-	-	-	-
**Gender (%)**			0.315	-	-	-
Female (ref)	39	21	-	-	-	-
Male	22	18	-	-	-	-
**Activity sector (%)**			0.699	-	-	-
Public (ref)	32	22	-	-	-	-
Private	29	17	-	-	-	-
**Experience length (%)**			0.118	-	-	-
˂ 10 years (ref)	31	26	-	-	-	-
≥ 10 years	30	13	-	-	-	-
**Training in diabetes management (%)**			˂ 0.001	5.635	2.238–14.190	˂ 0.001
Yes (ref)	39	9	-	-	-	-
No	22	30	-	-	-	-

Note: Participants over 50 years old and those who received training were significantly associated with a high level of knowledge. All variables in bivariate analysis with a *p*-value ˂ 0.05 have been included in multivariate logistic regression. In multivariate analysis, training in diabetes management was the only factor independently associated with primary healthcare knowledge level.

ref., reference value; CI, confidence interval.

In multivariate analysis, only training in diabetes management was significantly associated with primary health practitioners’ knowledge (OR: 5.635, 95% confidence interval [CI]: 2.238–14.19, *p* ˂ 0.001) (Table 3).

## Discussion

This study evaluated the knowledge level of Moroccan primary healthcare providers in the region of Marrakesh about DKD screening, management and referral guidelines, and determined its associated factors.

Our study population was young, with 67% of participants under 50 years of age, the majority were female, and 57% had less than 10 years of experience. Similarly, in a study conducted in Saudi Arabia evaluating clinical practices of primary care providers in DKD, the mean age of the participants was 35 years with a mean experience length of 7 years, but the proportion of male physicians was higher than female physicians (59%).^15^

Regarding screening for DKD, our findings have indeed revealed that more than half of the participants correctly identified that a positive albuminuria and reduced GFR were key indicators of DKD.

Compared to our results, an online survey of the proficiency of primary healthcare physicians in managing diabetes-related CKD showed that 52.4% of the participants were uncertain regarding their knowledge of the criteria for the diagnosis of DKD.^18^

Most of our participants reported that the ideal timing for first screening of DKD was at the time of diagnosis of type 2 diabetes, and that, essentially, albuminuria (97%) and, to a lesser extent, the GFR (51%) should be annually screened. However, our participants presented insufficient knowledge of the optimal timing for screening in patients with type 1 diabetes and the ideal test to use for screening for albuminuria. In a cross-sectional survey conducted among 160 Canadian physicians, including 60 general practitioners, the timing of CKD screening with patients in relation to type 2 diabetes diagnosis occurred for most physicians at diagnosis of type 2 diabetes (58.1%), using eGFR (100%) and UACR (96.8%).^19^ In the Saudi study, good knowledge was reported in terms of annual screening (77.4%), timely start of screening of DKD in patients with type 1 (52.1%) and type 2 (75.6%) diabetes and the use of UACR as a screening tool for DKD (85.5%).^15^

In our study, good scores were also reported about first-line recommended medications, but low scores were noted about the use of novel treatment (SGLT2i) and the timing of referral to a nephrologist. In a study of adult primary care patients with DKD, 80.3% had a care gap in SGLT2i prescription, and 42% had a care gap in RAASi prescription. These care gaps for SGLT2i and RAASi were especially observed in patients with DKD with well-controlled diabetes and blood pressure, suggesting failure to recognise DKD as an independent indication for these medications.^20^ On the contrary, the Saudi study showed high rates of prescription of ACE inhibitors (82.1%) and empagliflozin (73.9%) in addition to a good knowledge of indications of referral to a nephrologist (80.8%).^15^ In this Saudi study, higher levels of adherence to the guidelines were associated with the physicians’ education level, access to clinical guidelines and receipt of relevant training.^15^

Overall, the majority of our participants (61%) showed high levels of adherence to DKD guidelines, but our results also reflected varying degrees of knowledge across different aspects of DKD management. Our findings also revealed that higher levels of adherence to the guidelines were observed among physicians who received training in diabetes management. This training can be facilitated by setting up primary care kidney disease management programmes. Therefore, a multifactorial approach is necessary based on collaborative efforts to optimise the knowledge level of primary care providers in dealing with DKD.^21^ This lack of DKD knowledge among primary care providers may be one of the causes of delay in CKD identification and treatment, and may be because of many factors, such as the complexity and continuous evolution of the guidelines^20^ and the absence of communication between primary healthcare and nephrologists.^22^

The strengths of this study were that it provided an in-depth analysis of the knowledge level about DKD in Moroccan primary healthcare providers. However, there were some limitations. This study was limited by the small number of participants, its cross-sectional nature, and its reliance on self-reported data rather than a real-time evaluation of practices at primary care centres. Despite these limitations, we were able to identify shortcomings in the management of patients with DKD by general practitioners, which is relevant to running a comprehensive healthcare programme centred around DKD. The results of our study demonstrated the importance of continuous education and can guide the development of national programmes of training of primary healthcare providers in DKD in order to improve guideline compliance.

## Conclusion

This study highlighted unmet needs for care and management of DKD patients at the primary care level. It is well-known that in order to ensure the success of any renal healthcare intervention, it is imperative to effectively involve general practitioners using a well-structured approach. Such an approach will need proper training and the implementation of effective primary care disease programmes.

## Appendix 1

### Questionnaire:

#### • Section 1

Physician’s age:

Gender:

Activity sector:

Experience length:

Mean number of patients with diabetes consulting per month:

Training in diabetes management:

#### • Section 2

Evaluation of physicians’ knowledge level:

1. What are the diagnostic criteria of diabetic kidney disease (DKD)?
  - A positive albuminuria ± reduced glomerular filtration rate (GFR) in a patient with diabetes
  - An oedematous state
  - A positive haematuria
  - No idea
2. When should patients with type 2 diabetes first be screened for DKD?
  - At the time of diagnosis
  - 5 years after the onset of the disease
  - When the patient develops clinical signs of the disease
  - No idea
3. When should patients with type 1 diabetes first be screened for DKD?
  - At the time of diagnosis
  - 5 years after the onset of the disease
  - When the patient develops clinical signs of the disease
  - No idea
4. The diagnosis of DKD may be suspected if patients with diabetes present:
  - A single positive test of albuminuria
  - Two or three positive tests of albuminuria at an interval of 3–6 months
  - A haematuria
  - No idea
5. The recommended way to screen for albuminuria is:
  - A 24-h urine for albumin collection
  - A urine dipstick
  - A spot urinary albumin-to-creatinine ratio (UACR)
  - No idea
6. The minimal threshold for a positive albuminuria corresponds to:
  - UACR ≥ 30 mg/g
  - UACR > 150 mg/g
  - UACR > 300 mg/g
  - No idea
7. The recommended nephroprotective treatment of DKD:
  - Calcic inhibitors
  - Angiotensin-converting enzyme (ACE) inhibitors or angiotensin receptor blocker (ARB)
  - Beta-blockers
  - No idea
8. When should a general practitioner refer a patient with diabetes to a nephrologist?
  - As soon as a positive albuminuria is diagnosed
  - When GFR is < 30 mL/min on two consecutive visits or in case of a rapid decline of GFR
  - All above
  - No idea
9. What is the recommended target of arterial pressure if the patient presents with a positive albuminuria?
  - < 140/90 mmHg
  - < 130/80 mmHg
  - < 150/90 mmHg
  - No idea
10. What is the recommended target of HbA_1c_ test in a patient with diabetes and chronic kidney disease (CKD)?
  - ≤ 6.5%
  - ≤ 7%
  - ≤ 8%
  - No idea
11. GFR should be annually screened: True or False
12. Albuminuria should be annually screened: True or False
13. Metformin is contraindicated if GFR is:
  - <45 mL/min/1.73 m^2^
  - <60 mL/min/1.73 m^2^
  - <30 mL/min/1.73 m^2^
  - No idea
14. SGLT2 inhibitors are recommended in:
  - Patients with type 2 diabetes, CKD and GFR < 20 mL/min/1.73 m^2^
  - Patients with type 1 diabetes
  - Patients with type 2 diabetes, CKD and GFR ≥ 20 mL/min/1.73 m^2^
  - No idea
